# High quality draft genome sequence of *Janthinobacterium psychrotolerans* sp. nov., isolated from a frozen freshwater pond

**DOI:** 10.1186/s40793-017-0230-x

**Published:** 2017-01-19

**Authors:** Xianzhe Gong, Stig Skrivergaard, Benjamin Smed Korsgaard, Lars Schreiber, Ian P. G. Marshall, Kai Finster, Andreas Schramm

**Affiliations:** 1 Section for Microbiology, Department of Bioscience, Aarhus University, Aarhus, Denmark; 2 Center for Geomicrobiology, Department of Bioscience, Aarhus University, Aarhus, Denmark; 3 Stellar Astrophysics Center, Department of Physics and Astronomy, Aarhus University, Aarhus, Denmark

**Keywords:** *Janthinobacterium psychrotolerans*, Freshwater sediment, Low temperature, Denitrification, Fermentation

## Abstract

Strain S3-2^T^, isolated from sediment of a frozen freshwater pond, shares 99% 16S rRNA gene sequence identity with strains of the genus *Janthinobacterium*. Strain S3-2^T^ is a facultative anaerobe that lacks the ability to produce violacein but shows antibiotic resistance, psychrotolerance, incomplete denitrification, and fermentation. The draft genome of strain S3-2^T^ has a size of ~5.8 Mbp and contains 5,297 genes, including 115 RNA genes. Based on the phenotypic properties of the strain, the low *in silico* DNA-DNA hybridization (DDH) values with related genomes (<35%), and the low whole genome-based average nucleotide identity (ANI) (<86%) with other strains within the genus *Janthinobacterium,* we propose that strain S3-2^T^ is the type strain (= DSM 102223 = LMG 29653) of a new species within this genus. We propose the name *Janthinobacterium psychrotolerans* sp. nov. to emphasize the capability of the strain to grow at low temperatures.

**Electronic supplementary material:**

The online version of this article (doi:10.1186/s40793-017-0230-x) contains supplementary material, which is available to authorized users.

## Introduction

The genus *Janthinobacterium* includes Gram-negative, motile, aerobic rod-shaped bacteria, which were isolated from soil and aquatic environments. Production of violacein, a purple, water-insoluble, secondary metabolite, is a feature commonly found in this genus [1, 2]. Violacein has anti-bacterial, anti-viral, and anti-fungal properties [3], and has even been reported to protect frogs against fungal infection, when produced by the frog skin microbiota [4].

Strain S3-2^T^, which is affiliated with the genus *Janthinobacterium* was isolated from freshwater sediment while screening for denitrifying bacteria. However, strain S3-2^T^ has traits that unambiguously distinguish it from the other strains of the genus [2, 5, 6]. Among these traits is the ability of strain S3-2^T^ to grow at −3 °C, and to ferment different sugars. In contrast to the other strains, strain S3-2^T^ does not produce the violet pigment violacein, not even when grown on glycerol medium (20 g L^−1^) that induces violacein synthesis in other members of the genus *Janthinobacterium*. Here we present the genome of strain S3-2^T^ as well as its classification and phenotypic features. Taken together, these characteristics support the circumscription of S3-2^T^ as novel species, *Janthinobacterium psychrotolerans* sp. nov.

## Organism information

### Classification and features

Sediment was obtained from a small fresh water pond near Aarhus, Denmark (coordinates 56.182804 N, 10.176294 E); the pond was covered with a thick layer of ice at the time of sampling. Strain S3-2^T^ was isolated at room temperature under oxic conditions from a diluted sediment sample (3 g in 10 mL sterile water) by direct plating on TSB agar, containing 3 g tryptic soy broth (Scharlau Chemie S.A., Spain) L^−1^, 15 g agar L^−1^.

**Fig. 1:**
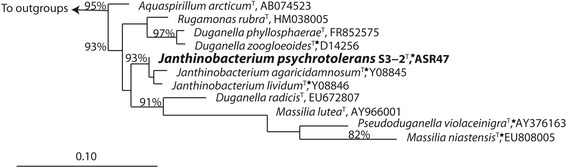
Phylogenetic relationship between *Janthinobacterium psychrotolerans* S3-2^T^ (shown in bold) and other closely related strains. The sequence alignment of 16S rRNA genes was generated using the Ribosomal Database Project (RDP) Aligner tool [32] and manually optimized with ARB [33]. The shown maximum likelihood tree was inferred based on the General Time Reversible (GTR) model with gamma rate heterogeneity as implemented in RAxML 7.4.2 [34]. Bootstrap support (1,000 replicates) of >70% is shown next to the branches. The genus *Polymucleobacter* was used to root the tree (not shown in the figure). Scale bar, 0.1 substitutions per nucleotide position. Star represents species with available sequenced genome in NCBI

Strain S3-2^T^ exhibits a 99% 16S rRNA sequence identity with *Janthinobacterium agaricidamnosum* (GenBank accession number: HG322949; IMG Genome ID 2585427668), the closest validly published species (Fig. 1).

Different growth temperatures (−3 °C, 0 °C, 4 °C, 10 °C, 21 °C, 25 °C, 30 °C, 35 °C, and 40 °C) were tested on TSB plates. Growth occurred between −3 °C and 30 °C, with the optimal growth temperature being 25 °C. The range of pH tolerance was tested in TSB (10 g L^−1^) adjusted to pH values 4–9 and buffered with citric acid, phosphate, or Tris [7]. Growth occurred between pH 6 and 8, with optimal growth at pH 7. Salt tolerance was tested on TSB (10 g L^−1^) agar with NaCl concentrations ranging from 0.17% to 3.17%. Strain S3-2^T^ tolerated up to 2.17% of NaCl. Strain S3-2^T^ produced N_2_O (determined by an N_2_O sensor [8]) as the end product of denitrification in anoxic incubations with TSB containing 5 mM nitrate; nitrite or N_2_ gas were never detected.

**Fig. 2:**
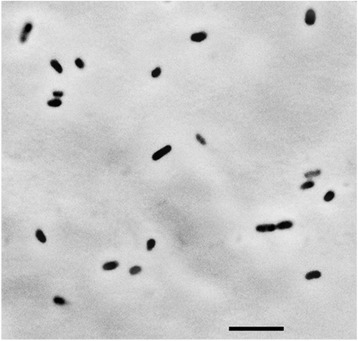
Phase contrast micrograph of *Janthinobacterium psychrotolerans* strain S3-2^T^. Scale bar, 5 μm

Cells of strain S3-2^T^ are rod-shaped, and stain Gram-negative. Cells in stationary growth phase on TSB agar were motile, and had a mean length of 1.9 ± 0.3 μm, and a mean width of 0.7 ± 0.1 μm under a phase contrast microscope (*n* = 27) (Fig. 2).

Strain S3-2^T^ showed mucoid pale yellow colonies on TSB agar, while colonies were non-mucoid, circular with undulate margins, and orange on modified Lysogeny broth (LB) agar (10 g L^−1^ tryptone, 5 g L^−1^ yeast extract, 10 g L^−1^ NaCl, 1% glycerol, 15 g L^−1^ agar), and brownish on glycerol medium (20 g L^−1^ glycerol, 0.5 g L^−1^ NaCl, 2.4 g L^−1^ MgSO_4_, 1 ml L^−1^ trace metal solution [9], 15 g L^−1^ agar). None of the media induced the production of violacein [10]. None of the observed pigments were fluorescent under UV light (365 nm; Vilber Lourmat, Germany).

Strain S3-2^T^ was resistant to penicillin (5 μg disc), and ampicillin (10 μg disc), but susceptible to streptomycin (10 μg disc) and tetracycline (30 μg disc) on TSB (3 g L^−1^) agar. In GEN III microplate assays (Biolog), strain S3-2^T^ was resistant to rifamycin SV, lincomycin, and vancomycin; susceptible to niaproof 4. Strain S3-2^T^ did not inhibit growth of *Escherichia coli* K12 (DSM498; a strain resistant to penicillin, ampicillin, streptomycin, and tetracycline) on TSB (10 g L^−1^) agar.

Strain S3-2^T^ was tested positive for alkaline phosphatase using the API ZYM test (BioMérieux, France), catalase using hydrogen peroxide, and oxidase (Bactident Oxidase, Merck, Germany). Using API 20E (BioMérieux, France), positive reactions were observed for enzymatic activity of arginine dihydrolase, for indole production, and the fermentation of D-glucose, D-mannitol, D-sucrose, and L-arabinose. Negative reactions were observed for enzymatic activities of β-galactosidase, lysine decarboxylase, ornithine decarboxylase, urease, and gelatinase. Inositol, D-sorbitol, L-rhamnose, D-melibiose, and amygdalin were not fermented, and H_2_S and acetoin were not produced. *Janthinobacterium* has previously been considered as non-fermentative [1, 11]. The capability of linking fermentation to growth has only been reported for *J. lividum* strain UTB1302 with glucose [5]. Using API 20NE (BioMérieux, France), positive reactions were observed for hydrolysis of esculin ferric citrate, and the assimilation of arabinose. Negative reactions were observed for the assimilation of D-maltose, phenylacetic acid, N-acetyl-glucosamine, capric acid, and adipic acid. According to GEN III microplate assays (Biolog) at 25 °C, strain S3-2^T^ could metabolize dextrin, D-cellobiose, D-raffinose, α-D-lactose, D-salicin, D-mannose, D-galactose, L-fucose, L-rhamnose, inosine, D-mannitol, D-arabitol, myo-inositol, glycyl-L-proline, L-alanine, L-aspartic acid, L-glutamic acid, L-histidine, L-pyroglutamic acid, D-galacturonic acid, L-galacturonic acid lactone, L-lactic acid, citric acid, α-keto-glutaric acid, D-malic acid, L-malic acid, bromo-succinic acid, Tween 40, and α-hydroxy-butyric acid. D-maltose, D-trehalose, N-acetyl-D-galactosamine, and formic acid were not metabolized.

The generation time of strain S3-2^T^ was approx. 160 min in TSB (10 g L^−1^) with 5 mM nitrate when grown aerobically at 20 °C. Overall, strain S3-2^T^ has traits that unambiguously distinguish it from other strains of the genus [2, 5, 6]. Among these traits is the ability of strain S3-2^T^ to grow at −3 °C. In contrast to the other strains, strain S3-2^T^ does not produce the pigment violacein, not even when grown on glycerol medium (20 g L^−1^), which induces violacein synthesis in other members of the genus *Janthinobacterium* [10]. Strain S3-2^T^ is available from the Belgian Co-ordinated Collection of Micro-organisms - BCCM/LMG Bacteria Collection as strain LMG 29653 and the Leibniz Institute DSMZ - German Collection of Microorganisms and Cell Cultures as strain DSM 102223; its general properties are summarized in Table 1.

**Table 1:** Classification and general features of *Janthinobacterium psychrotolerans* S3-2^T^ [35]

MIGS ID	Property	Term	Evidence code^a^
	Classification	Domain *Bacteria*	TAS [36]
		Phylum *Proteobacteria*	TAS [37]
		Class *Betaproteobacteria*	TAS [38]
		Order *Burkholderiales*	TAS [39]
		Family *Oxalobacteraceae*	TAS [40]
		Genus *Janthinobacterium*	TAS [40]
		Species *Janthinobacterium psychrotolerans*	TAS [40]
		Strain S3-2^T^ (LMG 29653 = DSM 102223)	IDA
	Gram stain	Negative	IDA
	Cell shape	Rod	IDA
	Motility	Motile	IDA
	Sporulation	None	IDA
	Temperature range	−3 °C – 30 °C	IDA
	Optimum temperature	25 °C	IDA
	pH range; Optimum	6–8; 7	IDA
	Carbon source	Sugars, amino acids, fatty acids etc.	IDA
MIGS-6	Habitat	Freshwater sediment	IDA
MIGS-6.3	Salinity	0.17–2.17% NaCl (w/v)	IDA
MIGS-22	Oxygen requirement	Facultative anaerobic	IDA
MIGS-15	Biotic relationship	Free-living	IDA
MIGS-14	Pathogenicity	Unknown	IDA
MIGS-4	Geographic location	Aarhus, Denmark	IDA
MIGS-5	Sample collection	2015–01-16	IDA
MIGS-4.1	Latitude	56°10'58.1"N	IDA
MIGS-4.2	Longitude	10°10'34.7"E	IDA
MIGS-4.4	Altitude	70 m	IDA

^a^Evidence codes - IDA: Inferred from Direct Assay; TAS: Traceable Author Statement (i.e., a direct report exists in the literature); NAS: Non-traceable Author Statement (i.e., not directly observed for the living, isolated sample, but based on a generally accepted property for the species, or anecdotal evidence). These evidence codes are from the Gene Ontology project [41]

## Genome sequencing information

### Genome project history

The draft genome sequence of strain S3-2^T^ was completed on December 21, 2015. The genome project is deposited in the Genomes OnLine Database (GOLD) as project Gp0124039. This Whole Genome Shotgun project has been deposited at GenBank under the accession LOCQ00000000. The version described in this paper is version LOCQ01000000. The summarized information of this project is shown in Table 2.

**Table 2:** Project information

MIGS ID	Property	Term
MIGS 31	Finishing quality	High quality draft
MIGS-28	Libraries used	NexteraXT DNA sample preparation
MIGS 29	Sequencing platforms	Illumina MiSeq
MIGS 31.2	Fold coverage	178
MIGS 30	Assemblers	SPAdes 3.6.1
MIGS 32	Gene calling method	Prodigal v2.6.2
	Locus Tag	ASR47
	Genbank ID	LOCQ00000000
	GenBank Date of Release	2017-01-31
	GOLD ID	Gp0124039
	BIOPROJECT	PRJNA300713
MIGS 13	Source Material Identifier	LMG 29653, DSM 102223
	Project relevance	Environmental, denitrification

### Growth conditions and genomic DNA preparation

Strain S3-2^T^ was grown at 25 °C in TSB (10 g L^−1^) supplemented with 5 mM nitrate. The cells were harvested by centrifugation and DNA was extracted from the pellet using the PowerLyser® PowerSoil® DNA extraction kit (MoBio, Carlsbad, CA, USA) according to the manufacturer’s protocol.

### Genome sequencing and assembly

The genome of strain S3-2^T^ was sequenced with the Illumina MiSeq Reagent Kit V3 (Illumina Inc. San Diego, CA, USA). Sequencing libraries were prepared using the Nextera XT Library Preparation Kit (Illumina). The sequencing library produced 3,761,645 paired end reads totalling ~2.11 Gbp. In total, 2,868,634 reads remained after quality trimming and adapter removal with Trimmomatic-0.33 [12] and the following trimming parameters: *CROP:235 HEADCROP:25 SLIDINGWINDOW:4:20*. Read quality before and after trimming was assessed by FastQC version 0.11.4 [13]. The trimmed reads (~1.04 Gbp) represented an average genome coverage of ~178-fold based on the size of the assembled draft genome of strain S3-2^T^. Reads were assembled using SPAdes 3.6.1 [14]. Contigs shorter than 1,000 bp were removed after the assembly.

### Genome annotation

The draft genome was annotated using the standard operation procedure of the DOE-JGI Microbial Genome Annotation Pipeline (MGAP v.4) supported by the JGI (Walnut Creek, CA; USA) [15]. Briefly, CRISPR elements were determined by the programs CRT [16] and PILER-CR v1.06 [17]. Non-coding RNAs, and tRNAs, were predicted by tRNAscan-SE 1.3.1 [18]. rRNA genes were identified by HMMER 3.1b2 [19]. Protein-coding genes were determined by Prodigal v2.6.2 [20]. Functional annotation was based on assigning the genes to different databases: the COG & KOG database (November, 2014) [21], the KEGG database (release 71.0, July 2014) [22], the MetaCyc database (release 18.1, June 2014) [23], the Pfam database (version 28.0, May, 2015) [24], the TIGRfam database (release 14.0, January, 2014) [25], and the InterPro Scan database (release 48) [26]. *In silico* DNA-DNA hybridization (GGDC 2.0) was carried out with the online genome-to-genome calculator provided by the DSMZ [27].

## Genome properties

The properties of the draft genome of strain S3-2^T^ are summarized in Table 3, and the assignment of genes into COG functional categories is shown in Table 4. The assembled draft genome features a G + C content of 63.04 mol%, and consists of 62 contigs ranging in size from 1,026 bp to 498,889 bp and totalling 5,844,062 bp. Based on CheckM 1.0.3 [28] in concert with conserved single copy genes detected in four reference genomes of *Janthinobacterium* spp. or 5,449 bacterial genomes, the genome of strain S3-2^T^ was estimated to be 98.28% or 95.69% complete, respectively. There are 5,182 (97.83%) protein-coding genes and 115 RNAs of the 5,297 predicted genes. Of the RNA, 77 are tRNAs and 25 are rRNAs. Based on the number of 5S, and partial 16S and 23S rRNA genes, the genome features at least 8 rRNA operons. Putative functions were assigned to 77.08% of the protein-coding genes.

**Table 3:** Genome statistics

Attribute	Value	% of Total^a^
Genome size (bp)	5,844,062	100
DNA coding (bp)	5,291,400	90.54
DNA G + C (bp)	3,683,895	63.04
DNA scaffolds	62	100
Total genes	5,297	100
Protein coding genes	5,182	97.83
RNA genes	115	2.17
Pseudo genes	0	0
Genes in internal clusters	639	12.06
Genes with function prediction	4,087	77.16
Genes assigned to COGs	3,543	66.89
Genes with Pfam domains	4,291	81.01
Genes with signal peptides	724	13.67
Genes with transmembrane helices	1,275	24.07
CRISPR repeats	0	0

**Table 4:** Number of genes associated with general COG functional categories

Code	Value	%age	Description
J	217	5.35	Translation, ribosomal structure and biogenesis
A	1	0.02	RNA processing and modification
K	338	8.33	Transcription
L	119	2.93	Replication, recombination and repair
B	2	0.05	Chromatin structure and dynamics
D	36	0.89	Cell cycle control, Cell division, chromosome partitioning
V	105	2.59	Defense mechanisms
T	336	8.28	Signal transduction mechanisms
M	268	6.61	Cell wall/membrane biogenesis
N	183	4.51	Cell motility
U	87	2.14	Intracellular trafficking and secretion
O	182	4.49	Posttranslational modification, protein turnover, chaperones
C	221	5.45	Energy production and conversion
G	256	6.31	Carbohydrate transport and metabolism
E	309	7.62	Amino acid transport and metabolism
F	87	2.14	Nucleotide transport and metabolism
H	190	4.68	Coenzyme transport and metabolism
I	174	4.29	Lipid transport and metabolism
P	232	5.72	Inorganic ion transport and metabolism
Q	87	2.14	Secondary metabolites biosynthesis, transport and catabolism
R	331	8.16	General function prediction only
S	233	5.74	Function unknown
-	1754	33.11	Not in COGs

The total is based on the total number of protein coding genes in the genome

## Insights from the genome sequence

There is a high 16S rRNA sequence identity of 99% between strain S3-2^T^ and all other strains with validly published names within the genus *Janthinobacterium* (Table 5). This value is higher than the 98.7% identity threshold recommended by Meier-Kolthoff et al. [29] to propose a new species within the phylum *Proteobacteria*. Therefore, *in silico* DNA-DNA hybridization (DDH) against reference genomes was performed using the online genome-to-genome calculator with the GGDC 2.0 BLAST+ model [27]. DDH values calculated according to formula 2 (to confidently predict DDH values of incomplete genomes [27]) were always <35% (Table 5, and Additional file 1: Table S1), and thus clearly below the 70% threshold to differentiate bacterial species [27]. Whole-genome-based average nucleotide identities (ANI) to other *Janthinobacterium* genomes were calculated by the online tool [30, 31]. ANI was always < 86% (Table 5, and Additional file 1: Table S2) [30], and thus also well below the threshold for species delineation (95%; [31]). Hence, both results support the phenotypic distinction of strain S3-2^T^ as novel species within the genus *Janthinobacterium*.

**Table 5:** Sequence similarity of *J. psychrotolerans* strain S3-2^T^ with described species of the genus *Janthinobacterium*

Strain name	16S rRNA identity (%)^a^	DDH (Model-based Confidence Interval) (%)	ANI ± SD^b^ (%)
*Janthinobacterium agaricidamnosum*	99	23.3 (21.0–25.7)	81.66 ± 5.07
*Janthinobacterium lividum* MTR	98	28.0 (25.6–30.5)	84.69 ± 4.73
*Janthinobacterium lividum* NFR18	99	27.7 (25.3–30.2)	84.73 ± 4.87
*Janthinobacterium lividum* PMC 25724	99	26.6 (24.3–29.1)	83.84 ± 4.58
*Janthinobacterium lividum* RIT308	99	27.9 (25.5–30.4)	84.75 ± 4.84

^a^Values for 16S rRNA identity are based on BLAST of the S3-2^T^ 16S rRNA gene against genomes in IMG, except for *J. lividum* MTR, which was retrieved from NCBI (acc. nr. JQ070957.1)

^b^SD: Standard Deviation

Violacein production, a common feature in *Janthinobacterium*, was never observed in growth studies with strain S3-2^T^. This observation is consistent with the absence of the *vioABCDE* operon, which encodes the genes required for the synthesis of this pigment; neither the automated annotation nor manual BLAST searches of the S3-2^T^ genome for known components of the *vioABCDE* operon (Additional file 1: Table S3) [2, 6] identified any genes encoding violacein synthesis.

The genome of strain S3-2^T^ features all necessary genes for nitrate reduction to N_2_O but lacks genes encoding the nitrous oxide reductase (Additional file 1: Table S4), which is consistent with N_2_O as end-product of denitrification. Genes affiliated with aerobic respiration were identified, including terminal oxidases with both high- and low-affinity for oxygen (Additional file 1: Table S5). Another characteristic of strain S3-2^T^ is its capability to ferment different sugars, a trait which has not been reported for other strains in the genus *Janthinobacterium* [1, 5, 11]. The genes that encode these properties were summarized (Additional file 1: Table S6, and Figure S1).

## Conclusions

Based on the phenotypic properties, phylogenetic position, and whole genome comparison, we formally propose strain S3-2^T^ as novel species of the genus *Janthinobacterium*, for which we propose the name *Janthinobacterium psychrotolerans* sp. nov. with strain S3-2^T^ (=DSM 102223 = LMG 29653) as the type strain.

## Description of *Janthinobacterium psychrotolerans* sp. nov.

*Janthinobacterium psychrotolerans* (psy.chro.to'le.rans. Gr. adj. psychros cold; L. part. adj. tolerans tolerating; N.L. neut. part. adj. *psychrotolerans* tolerating cold temperatures).

*Janthinobacterium psychrotolerans* is a facultative anaerobic, Gram-negative bacterium. Cells are rod-shaped, motile, and have a size of 1.9 ± 0.3 × 0.7 ± 0.1 μm. Colonies are pale yellow and mucoid on TSB agar. Growth occurs between −3 and 30 °C, with optimal growth observed at 25 °C. Strain S3-2^T^ tolerates salinity between 0.17% and 2.17% NaCl, and grows within the pH range of 6 to 8 with optimal growth observed at pH 7.

Positive for catalase, oxidase, alkaline phosphatase, arginine dihydrolase. Negative for β-galactosidase, lysine decarboxylase, ornithine decarboxylase, urease, gelatinase.

Positive for metabolizing dextrin, D-cellobiose, D-raffinose, α-D-lactose, D-salicin, D-mannose, D-galactose, L-fucose, L-rhamnose, inosine, D-mannitol, D-arabitol, myo-inositol, glycyl-L-proline, L-alanine, L-aspartic acid, L-glutamic acid, L-histidine, L-pyroglutamic acid, D-galacturonic acid, L-galacturonic acid lactone, L-lactic acid, citric acid, α-keto-glutaric acid, D-malic acid, L-malic acid, bromo-succinic acid, Tween 40, and α-hydroxy-butyric acid. Negative for metabolizing D-maltose, D-trehalose, N-acetyl-D-galactosamine, and formic acid.

Positive for hydrolysis of esculin ferric citrate, assimilation of arabinose, and indole production. Negative for assimilation of D-maltose, phenylacetic acid, N-acetyl-glucosamine, capric acid, and adipic acid, acetoin production, and H_2_S production.

Strain S3-2^T^ is able to ferment D-glucose, D-mannitol, D-sucrose, and L-arabinose; unable to ferment inositol, D-sorbitol, L-rhamnose, D-meliblose, and amygdalin.

Resistant to penicillin, vancomycin, rifamycin SV, lincomycin, and ampicillin; susceptible to streptomycin, niaproof 4, and tetracycline.

The G + C content of the genome is 63.04 mol%. The genome project is deposited in the Genomes OnLine Database (GOLD) as project Gp0124039. This Whole Genome Shotgun project is deposited at GenBank under the accession LOCQ00000000. The type strain S3-2^T^ (= LMG 29653 = DSM 102223) was isolated from sediment of a small, frozen pond in Hasle, Aarhus, Denmark (coordinates 56.182804 N, 10.176294 E) in January, 2015.

## Additional file

Additional file 1: Table S1. Results of *in silico* DNA-DNA hybridization (DDH) of the assembled strain S3-2 draft genome against all published *Janthinobacterium* genomes using the online genome-to-genome calculator with the GGDC 2.0 BLAST+ model [1]. Displayed values were calculated according to formula 2, the only formula able to confidently predict DDH values of incomplete genomes [1]. The threshold to delineate two distinct species is 70% [1]. **Table S2.** Whole genome-based average nucleotide identity (ANI) of strain S3-2 to other sequenced *Janthinobacterium* genomes [2, 3]. The threshold to delineate two distinct species is 95% [3]. **Table S3.** Locus tags of the *vioABCDE* operon in other *Janthinobacterium* genomes in IMG. **Table S4.** Genomic inventory for denitrification in strain S3-2 based on the annotation from IMG. **Table S5.** Genomic inventory for terminal oxidases in strain S3-2 based on the annotation from IMG. **Table S6.** Genes encoding the D-Glucose fermentation pathway in strain S3-2 based on the annotation from IMG. **Figure S1.** Pathway of D-Glucose fermentation in strain S3-2 based on the annotation from IMG. (1), glucokinase; (2), glucose-6-phosphate isomerase; (3), 6-phosphofructokinase 1; (4), fructose-bisphosphate aldolase; (5), triosephosphate isomerase; (6), glyceraldehyde 3-phosphate dehydrogenase; (7), phosphoglycerate kinase; (8), 2,3-bisphosphoglycerate-dependent phosphoglycerate mutase; (9), probable phosphoglycerate mutase; (10), enolase; (11, 12), pyruvate kinase; (13, 14), pyruvate dehydrogenase (quinone); (15, 16), L-lactate dehydrogenase (cytochrome). For gene details, see Table S5. (DOCX 41 kb)

## Abbreviations

ANI: Average nucleotide identities
DDH: DNA-DNA hybridization
GOLD: Genomes OnLine database
GTR: General time reversible
LB: Lysogeny broth
MGAP: Microbial genome annotation pipeline
RDP: Ribosomal database project
TSB: Tryptic soy broth.
