# Supplementary material for: High quality draft genome sequence of Janthinobacterium psychrotolerans sp. nov., isolated from a frozen freshwater pond
Source: Stand Genomic Sci. 2017 Jan 19;12:8. doi: 10.1186/s40793-017-0230-x (PMC5244535; doi:10.1186/s40793-017-0230-x)
Supplement: Additional file 1: Table S1. — Results of in silico DNA-DNA hybridization (DDH) of the assembled strain S3-2 draft genome against all published Janthinobacterium genomes using the online genome-to-genome calculator with the GGDC 2.0 BLAST+ model [1]. Displayed values were calculated according to formula 2, the only formula able to confidently predict DDH values of incomplete genomes [1]. The threshold to delineate two distinct species is 70% [1]. Table S2. Whole genome-based average nucleotide identity (ANI) of strain S3-2 to other sequenced Janthinobacterium genomes [2, 3]. The threshold to delineate two distinct species is 95% [3]. Table S3. Locus tags of the vioABCDE operon in other Janthinobacterium genomes in IMG. Table S4. Genomic inventory for denitrification in strain S3-2 based on the annotation from IMG. Table S5. Genomic inventory for terminal oxidases in strain S3-2 based on the annotation from IMG. Table S6. Genes encoding the D-Glucose fermentation pathway in strain S3-2 based on the annotation from IMG. Figure S1. Pathway of D-Glucose fermentation in strain S3-2 based on the annotation from IMG. (1), glucokinase; (2), glucose-6-phosphate isomerase; (3), 6-phosphofructokinase 1; (4), fructose-bisphosphate aldolase; (5), triosephosphate isomerase; (6), glyceraldehyde 3-phosphate dehydrogenase; (7), phosphoglycerate kinase; (8), 2,3-bisphosphoglycerate-dependent phosphoglycerate mutase; (9), probable phosphoglycerate mutase; (10), enolase; (11, 12), pyruvate kinase; (13, 14), pyruvate dehydrogenase (quinone); (15, 16), L-lactate dehydrogenase (cytochrome). For gene details, see Table S5. (DOCX 41 kb) [file 40793_2017_230_MOESM1_ESM.docx]

**Xianzhe Gong, Stig Skrivergaard, Benjamin Smed Korsgaard, Lars Schreiber, Ian P.G. Marshall, Kai Finster and Andreas Schramm**

High qualify draft genome sequence of *Janthinobacterium psychrotolerans sp. nov.,* isolated from a frozen freshwater pond

**Supplementary materials**

**Table S1.** Results of *in silico* DNA-DNA hybridization (DDH) of the assembled strain S3-2 draft genome against all published *Janthinobacterium* genomes using the online genome-to-genome calculator with the GGDC 2.0 BLAST+ model [1]. Displayed values were calculated according to formula 2, the only formula able to confidently predict DDH values of incomplete genomes [1]. The threshold to delineate two distinct species is 70% [1].

| **Strain name** | **DDH (%)** | **Model-based Confidence Interval (%)** |
| --- | --- | --- |
| *Janthinobacterium agaricidamnosum* | 23.3 | 21.0 – 25.7 |
| *Janthinobacterium lividum* MTR | 28.0 | 25.6 – 30.5 |
| *Janthinobacterium lividum* NFR18 | 27.7 | 25.3 – 30.2 |
| *Janthinobacterium lividum* PMC 25724 | 26.6 | 24.3 – 29.1 |
| *Janthinobacterium lividum* RIT308 | 27.9 | 25.5 – 30.4 |
| *Janthinobacterium* sp. 344 | 27.9 | 25.6 – 30.4 |
| *Janthinobacterium* sp. 551a | 27.9 | 25.6 – 30.4 |
| *Janthinobacterium* sp. Ant5-2 | 31.1 | 28.7 – 33.6 |
| *Janthinobacterium* sp. Ant5-2-1 | 28.0 | 25.6 – 30.5 |
| *Janthinobacterium* sp. B9-8 | 34.7 | 32.2 – 37.2 |
| *Janthinobacterium* sp. CG3 | 23.4 | 21.1 – 25.8 |
| *Janthinobacterium* sp. CG23 2 | 21.4 | 19.1 – 23.8 |
| *Janthinobacterium* sp. HH01 | 22.1 | 19.8 – 24.6 |
| *Janthinobacterium* sp. KBS0711 | 27.8 | 25.5 – 30.3 |
| *Janthinobacterium* sp. Marseille | 19.8 | 17.6 – 22.3 |
| *Janthinobacterium* sp. OK676 | 28.0 | 25.6 – 30.4 |
| *Janthinobacterium* sp. RA13 | 28.0 | 25.6 – 30.5 |

**Table S2.** Whole genome-based average nucleotide identity (ANI) of strain S3-2 to other sequenced *Janthinobacterium* genomes [2,3]. The threshold to delineate two distinct species is 95% [3].

| **Strain name** | **ANI** ± **SD^1^ (%)** |
| --- | --- |
| *Janthinobacterium agaricidamnosum* | 81.66 ± 5.07 |
| *Janthinobacterium lividum* MTR | 84.69 ± 4.73 |
| *Janthinobacterium lividum* NFR18 | 84.73 ± 4.87 |
| *Janthinobacterium lividum* PMC 25724 | 83.84 ± 4.58 |
| *Janthinobacterium lividum* RIT308 | 84.75 ± 4.84 |
| *Janthinobacterium* sp. 344 | 84.75 ± 4.79 |
| *Janthinobacterium* sp. 551a | 84.74 ± 4.72 |
| *Janthinobacterium* sp. Ant5-2 | 85.71 ± 4.36 |
| *Janthinobacterium* sp. Ant5-2-1 | 84.58 ± 4.71 |
| *Janthinobacterium* sp. B9-8 | IH^2^ |
| *Janthinobacterium* sp. CG3 | 81.53 ± 4.70 |
| *Janthinobacterium* sp. CG23 2 | 79.53 ± 4.83 |
| *Janthinobacterium* sp. HH01 | 80.40 ± 4.91 |
| *Janthinobacterium* sp. KBS0711 | 84.68 ± 4.80 |
| *Janthinobacterium* sp. Marseille | 77.51 ± 4.39 |
| *Janthinobacterium* sp. OK676 | 84.79 ± 4.88 |
| *Janthinobacterium* sp. RA13 | 84.71 ± 4.84 |
| ^1^ SD: Standard Deviation.  ^2^ IN: Insufficient Hits to estimate ANI. |  |

**Table S3.** Locus tags of the *vioABCDE* operon in other *Janthinobacterium* genomes in IMG.

| **Strain name** | ***vioA*** | ***vioB*** | ***vioC*** | ***vioD*** | ***vioE*** |
| --- | --- | --- | --- | --- | --- |
| *Janthinobacterium agaricidamnosum* | GJA_1080 | GJA_1081 | GJA_1082 | GJA_1083 | GJA_1084 |
| *Janthinobacterium lividum* NFR18 | Ga0058997_1568 | Ga0058997_1567 | Ga0058997_1566 | Ga0058997_1565 | Ga0058997_1564 |
| *Janthinobacterium lividum* RIT308 | BW37_04193 | BW37_04192 | BW37_04191 | BW37_04190 | BW37_04189 |
| *Janthinobacterium* sp. B9-8 | Ga0077285_11839 | Ga0077285_11838 | Ga0077285_11837 | Ga0077285_11836 | Ga0077285_11835 |
| *Janthinobacterium* sp. HH01 | Jab_2c08810 | Jab_2c08820 | Jab_2c08830 | Jab_2c08840 | Jab_2c08850 |
| *Janthinobacterium* sp. RA13 | FG13DRAFT_1275 | FG13DRAFT_1276 | FG13DRAFT_1277 | FG13DRAFT_1278 | FG13DRAFT_1279 |

**Table S4.** Genomic inventory for denitrification in strain S3-2 based on the annotation from IMG.

| **Function** | **Gene** | **Locus Tag** | **GenBank Identifier (pBLAST best hit)** | **%Identity** |
| --- | --- | --- | --- | --- |
| Cytoplasmic dissimilatory nitrate reductase | *narG* | Ga0101933_1004191 | WP_008449123.1 *Janthinobacterium* sp. HH01 | 94 |
|  | *narH* | Ga0101933_1004190 | WP_008449121.1 *Janthinobacterium* sp. HH01 | 96 |
|  | *narI* | Ga0101933_1004188 | WP_036244233.1 *Massilia* sp. BSC265 | 85 |
|  | *narJ* | Ga0101933_1004189 | WP_008449120.1 *Janthinobacterium* sp. HH01 | 80 |
| Regulation of cytoplasmic dissimilatory nitrate reductase | *narL1* | Ga0101933_1004184 | WP_034781594.1 *Janthinobacterium* | 98 |
|  | *narL2* | Ga0101933_100990 | WP_046685279.1 *Janthinobacterium* | 90 |
| Nitrate/Nitrite transporter | *narK1* | Ga0101933_100144 | WP_020656744.1 *Massilia niastensis* | 77 |
|  | *narK2* | Ga0101933_1004194 | WP_046685044.1 *Janthinobacterium* sp. KBS0711 | 96 |
|  | *narK3* | Ga0101933_1004195 | WP_035818386.1 *Janthinobacterium* sp. RA13 | 94 |
|  | *narK4* | Ga0101933_1006105 | WP_035820112.1 *Janthinobacterium* sp. RA13 | 96 |
| Nitrite reductase (NO-forming) | *nirK* | Ga0101933_1003120 | WP_046684252.1 *Janthinobacterium* sp. KBS0711 | 85 |
| Dissimilatory nitric oxide reductase | *norB* | Ga0101933_1003122 | WP_035822175.1 Janthinobacterium sp. RA13 | 93 |

**Table S5.** Genomic inventory for terminal oxidases in strain S3-2 based on the annotation from IMG.

| **Oxygen affinity** | **Product name** | **Locus Tag** | **GenBank Identifier (pBLAST best hit)** | **%Identity** |
| --- | --- | --- | --- | --- |
| High-affinity | cytochrome c oxidase cbb3-type subunit 1 | Ga0101933_1010200 | WP_010393924.1 *Janthinobacterium lividum* | 97 |
|  | cytochrome c oxidase cbb3-type subunit 2 | Ga0101933_1010199 | WP_034752513.1 *Janthinobacterium lividum* | 97 |
|  | cytochrome c oxidase cbb3-type subunit 3 | Ga0101933_1010197 | WP_046681778.1 *Janthinobacterium* sp. KBS0711 | 92 |
|  | cytochrome c oxidase cbb3-type subunit 4 | Ga0101933_1010198 | WP_046681777.1 *Janthinobacterium* sp. KBS0711 | 85 |
| Low-affinity | cytochrome c oxidase subunit 1 | Ga0101933_1008163 | WP_034784874.1 *Janthinobacterium* | 98 |
|  | cytochrome c oxidase subunit 2 | Ga0101933_1008162 | WP_034784872.1 *Janthinobacterium lividum* | 96 |
|  | cytochrome c oxidase subunit 3 | Ga0101933_1008167 | WP_034748999.1 *Janthinobacterium* | 92 |
|  | cytochrome c oxidase subunit 1 | Ga0101933_1003327 | WP_047825711.1 *Massilia* | 83 |
|  | cytochrome c oxidase subunit 2 | Ga0101933_1003326 | KFC62340.1 *Massilia* sp. LC238 | 78 |
|  | cytochrome c oxidase subunit I+III | Ga0101933_1001163 | WP_056147649.1 *Duganella* sp. Leaf61 | 75 |
|  | cytochrome c oxidase subunit 2 | Ga0101933_1001164 | WP_036236422.1 *Massilia* sp. JS1662 | 62 |
| Low-affinity | cytochrome o ubiquinol oxidase subunit 4^(1)^ | Ga0101933_100752 | WP_010398411.1 *Janthinobacterium lividum* | 85 |
|  | cytochrome o ubiquinol oxidase subunit 3 | Ga0101933_100753 | WP_034788486.1 *Janthinobacterium* | 92 |
|  | cytochrome bo3 quinol oxidase subunit 1^(2)^ | Ga0101933_100754 | WP_046682725.1 *Janthinobacterium* | 96 |
|  | cytochrome o ubiquinol oxidase subunit 2 | Ga0101933_100755 | KKO64591.1 *Janthinobacterium* sp. KBS0711 | 92 |

(1): Annotated as cytochrome o ubiquinol oxidase operon protein in IMG.

(2): Annotated as cytochrome bo3 quinol oxidase subunit 1 apoprotein in IMG

**Table S6.** Genes encoding the D-Glucose fermentation pathway in strain S3-2 based on the annotation from IMG.

| **Locus Tag** | **Gene Product Name** | **GenBank Identifier (pBLAST best hit)** | **% Identity** |
| --- | --- | --- | --- |
| Ga0101933_101334 | glucokinase | WP_035825187.1 *Janthinobacterium* sp. RA13 | 91 |
| Ga0101933_1017109 | glucose-6-phosphate isomerase | WP_035828017.1 *Janthinobacterium* sp. RA13 | 92 |
| Ga0101933_101675 | 6-phosphofructokinase 1 | WP_034746054.1 *Janthinobacterium lividum* | 95 |
| Ga0101933_101651 | fructose-bisphosphate aldolase | WP_056150645.1 *Duganella* sp. Leaf126 | 97 |
| Ga0101933_1001361 | triosephosphate isomerase | WP_043462357.1 *Janthinobacterium lividum* | 89 |
| Ga0101933_101350 | glyceraldehyde 3-phosphate dehydrogenase | WP_010395244.1 *Janthinobacterium* | 98 |
| Ga0101933_101653 | phosphoglycerate kinase | WP_035822498.1 *Janthinobacterium* sp. RA13 | 97 |
| Ga0101933_1002326 | 2,3-bisphosphoglycerate-dependent phosphoglycerate mutase | WP_035823826.1 *Janthinobacterium* sp. RA13 | 96 |
| Ga0101933_100883 | probable phosphoglycerate mutase | WP_035824585.1 *Janthinobacterium* sp. RA13 | 90 |
| Ga0101933_100150 | enolase | WP_010400536.1 *Janthinobacterium lividum* | 98 |
| Ga0101933_1007205 | pyruvate kinase | WP_035827421.1 *Janthinobacterium* sp. RA13 | 96 |
| Ga0101933_101652 | pyruvate kinase | WP_046684386.1 *Janthinobacterium* | 94 |
| Ga0101933_1004139 | pyruvate dehydrogenase (quinone) | WP_026922676.1 *Glycomyces arizonensis* | 87 |
| Ga0101933_101696 | pyruvate dehydrogenase (quinone) | KQM34811.1 *Rhizobium* sp. Leaf202 | 80 |
| Ga0101933_1002110 | L-lactate dehydrogenase (cytochrome) | WP_057721457.1 *Pseudomonas orientalis* | 86 |
| Ga0101933_100645 | L-lactate dehydrogenase (cytochrome) | WP_035820051.1 *Janthinobacterium* sp. RA13 | 96 |

**Figure S1.** Pathway of D-Glucose fermentation in strain S3-2 based on the annotation from IMG. (1), glucokinase; (2), glucose-6-phosphate isomerase; (3), 6-phosphofructokinase 1; (4), fructose-bisphosphate aldolase; (5), triosephosphate isomerase; (6), glyceraldehyde 3-phosphate dehydrogenase; (7), phosphoglycerate kinase; (8), 2,3-bisphosphoglycerate-dependent phosphoglycerate mutase; (9), probable phosphoglycerate mutase; (10), enolase; (11, 12), pyruvate kinase; (13, 14), pyruvate dehydrogenase (quinone); (15, 16), L-lactate dehydrogenase (cytochrome). For gene details, see Table S5.

**Supplementary References:**

1. Meier-Kolthoff JP, Auch AF, Klenk H-P, Göker M. Genome sequence-based species delimitation with confidence intervals and improved distance functions. BMC Bioinformatics 2013;14:60.

2. Goris J, Konstantinidis KT, Klappenbach JA, Coenye T, Vandamme P, Tiedje JM. DNA-DNA hybridization values and their relationship to whole-genome sequence similarities. Int. J. Syst. Evol. Microbiol. 2007;57:81–91.

3. Rodriguez-R LM, Konstantinidis KT. Bypassing cultivation to identify bacterial species. Microbe Mag. 2014;9:111–8.
